# Efficacy of the biocontrol agent *Trichoderma hamatum* against *Lasiodiplodia theobromae* on macadamia

**DOI:** 10.3389/fmicb.2022.994422

**Published:** 2022-08-31

**Authors:** Xiaojiao Li, Jinsong Leng, Longfeng Yu, Haidong Bai, Xiaojun Li, Michael Wisniewski, Jia Liu, Yuan Sui

**Affiliations:** ^1^School of Biotechnology and Bioengineering, West Yunnan University, Lincang, China; ^2^Chongqing Key Laboratory of Economic Plant Biotechnology, College of Landscape Architecture and Life Science/Institute of Special Plants, Chongqing University of Arts and Sciences, Yongchuan, China; ^3^Lincang Academy of Forestry, Lincang, China; ^4^Department of Biological Sciences, Virginia Polytechnic Institute and State University, Blacksburg, VA, United States

**Keywords:** biological control, *Lasiodiplodia theobromae*, macadamia, *Trichoderma hamatum*, fungal disease

## Abstract

Macadamia (*Macadamia integrifolia*) trees are an important source of revenue in rainforest ecosystems. Their nuts are rich in vitamins, minerals, fiber, antioxidants, and monounsaturated oils. The fungus *Lasiodiplodia theobromae*, however, is a major disease problem, causing kernel rot and other disease symptoms. In the present study, a dual confrontation assay was used to evaluate the inhibitory effect of an endophytic strain of *Trichoderma hamatum* C9 from macadamia root against *L. theobromae*. Volatiles and cell-free culture filtrate of *T. hamatum* were also used to assess their antifungal activity against *L. theobromae*. Results suggested that *T. hamatum* exhibited a significant inhibitory effect against *L. theobromae in vitro*. Further results of a biocontrol assay indicated that a spray treatment of *T. hamatum* conidial suspension significantly decreased the size of lesions caused by artificially inoculated *L. theobromae* on macadamia leaves, as well as the disease index in young trees inoculated with *L. theobromae*, relative to sterile water controls. Collectively, our findings indicate that *T. hamatum* C9 represents a potential biocontrol agent that can be used to manage *L. theobromae* on macadamia.

## Introduction

Macadamia (*Macadamia integrifolia*) is an evergreen tree native to rainforest regions of southeastern Australia (Trueman, 2013). Over the past century, macadamia nuts have become an important internationally traded product (Carr, 2013). They have a high content of oil (69–78 g per 100 g fresh weight) and a relatively low percentage of saturated fatty acids. The consumption of oils with low levels of saturated fatty acids helps to improve blood lipid profiles and decreases inflammation and oxidative stress, thus, contributing to lowering body mass and generally reducing risk factors associated with cardiovascular disease (Aquino-Bolaños et al., 2017). Moreover, macadamia kernels are a rich source of tocotrienols and squalene, which are also considered to be nutraceuticals, and macadamia oil is a common product obtained from the processing of macadamia nuts (Wall, 2010; Navarro and Rodrigues, 2016).

The introduction and trial planting of macadamia in China began in the 1970s, and now macadamia is cultivated in the provinces of Guangdong, Yunnan, Guangxi, and Guizhou. The planted area of macadamia in China exceeded 301,206 hm^2^ by the end of 2018, and now China has the largest and fastest growing macadamia industry, accounting for over 1/3 of global production acreage (Shuai et al., 2022). Macadamia plants have been reported to be susceptible to a variety of fungal pathogens that can variously infect flower, leaf, fruit, stem, and root tissues (Akinsanmi and Drenth, 2006, 2017; Akinsanmi et al., 2016a,b; Prasannath et al., 2021a,b; Li et al., 2022). In this regard, species in the genus *Lasiodiplodia* represent a serious concern for the crop. *Lasiodiplodia* sp. (Akinsanmi and Drenth, 2017) and *Lasiodiplodia pseudotheobromae* (Chang et al., 2019) are responsible for causing husk rot resulting in diffuse soft and spongy black lesions on the fruit pericarp. *Lasiodiplodia theobromae* can also cause trunk cankers and shoot necrosis (Fischer et al., 2017). It has been reported that *L. theobromae* has a wide host range and geographical distribution, particularly in tropical and subtropical regions (Salvatore et al., 2020). Its pycnidia are stromatic, globose, and ostiolate. Conidia are initially hyaline, 1-celled and subovoid. When mature, typical conidia are 1-septate, brown and measured 26–31 × 12–16 μm (Fischer et al., 2017). Although macadamia cultivation still relies on the use of synthetic chemicals like carbendazim and pyraclostrobin (Akinsanmi et al., 2008; Khun et al., 2021) to minimize disease problems, there is a broad trend to explore and develop biocontrol agents, including the use of beneficial fungal endophytes, to manage tree diseases (Sosso et al., 2021).

Different species and strains of *Trichoderma* have been extensively studied and employed as biocontrol agents, due to their ubiquitous presence in soils, high efficacy, and established regulatory approval (Alghuthaymi et al., 2022). *Trichoderma* has been reported to be a dominant component of various soil mycobiomes, as well as a common fungal endophyte with biocontrol potential and plant growth promotion activity (Castro-Restrepo et al., 2022; Siebatcheu et al., 2022; Tyśkiewicz et al., 2022). Thus, *Trichoderma* has been widely used as a component of environmentally friendly agricultural management practices (Zin and Badaluddin, 2020). In this regard, *Trichoderma hamatum* has been recognized for its ability to induce systemic resistance in host plants and secrete antifungal compounds (Shaw et al., 2016; Abdelkhalek et al., 2022). It is known *Trichoderma* serves as a producer of volatile organic compounds; in particular, 6-n-pentyl-2H-pyran-2-one (6-PAP) (Jeleń et al., 2014) is very considered recently as a determinant of effects in plant protection. Studies utilizing *Trichoderma* as a biocontrol agent to manage macadamia tree diseases, however, are limited. The main objective of the present study was to evaluate the ability of *T. hamatum* to inhibit *L. theobromae in vitro*, as well to limit disease on macadamia leaves and whole plants. The antifungal activity of volatiles and cell-free culture filtrate of *T. hamatum* against *L. theobromae* was also assessed.

## Materials and methods

### Biocontrol and pathogenic fungi

**FIGURE 1:**
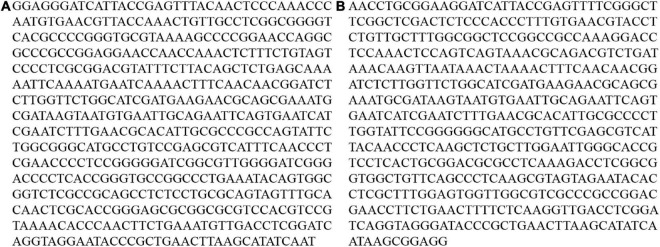
The partial nucleotide sequences of ITS rDNA of the strain C9 **(A)** and the strain L1 **(B)** obtained in our study.

The endophytic fungus, *T. hamatum* strain C9, was originally isolated in our laboratory from a root of a healthy macadamia tree (*M. integrifolia* × *M. tetraphylla* hybrid cv. A4) growing in a major production area in Lincang City, Yunnan Province, China (24°1′-24°11′N, 99°33′-99°43′E). The fungal pathogen, *L. theobromae* strain L1, was originally isolated from a root of an infected macadamia tree growing in the same region. Both fungal isolates were identified based on their morphology and the nucleotide sequence of ITS rDNA (Malachová et al., 2020; Baazeem et al., 2021). Specifically, the partial nucleotide sequences of ITS rDNA of the strain C9 (Figure 1A) and the strain L1 (Figure 1B) obtained in our study were 100% identical to those of *T. hamatum* isolate F4 (NCBI Accession: MT341773.1) and *L. theobromae* isolate FH14K03 (NCBI Accession: MK886711.1), respectively. Both fungi were cultured on PDA (potato dextrose agar) and grown at 25°C prior to use.

### Plant material

Macadamia (*M. integrifolia* × *M. tetraphylla* hybrid cv. A4) plants were purchased in September 2021, from suppliers in the macadamia production area where the fungi were collected. Three-year-old plants with a height of 1 – 1.3 m and fully expanded leaves from the top third of the plants were used in this study.

### Dual confrontation assay

A dual confrontation assay was used to quantify the interaction between *T. hamatum* C9 and *L. theobromae* L1 *in vitro* (Stracquadanio et al., 2020). Mycelial disks (7 mm in diameter) obtained from the margins of 5-day-old PDA cultures of *T. hamatum* and *L. theobromae* were placed on opposite sides (50-mm distance) of a 90-mm PDA plate. The PDA plates were then incubated at 25°C for 72 h. The radial growth of *L. theobromae* (the smallest colony diameter) was then measured in the presence (treatment) or absence (control) of *T. hamatum*. Three biological replicates were used in each assay and the assay was repeated three times.

### Assessment of *Trichoderma hamatum* C9 cell-free culture filtrate and volatiles on the growth of *Lasiodiplodia theobromae* L1

A conidial suspension of *T. hamatum* was obtained from 5-day-old PDA cultures and adjusted to a concentration of 1 × 10^6^ spores/ml that was quantified with a hemocytometer. Then, 100 μl of the conidial suspension was added to 100 ml of PDB and the inoculated broth was incubated for 3 day at 25°C on a rotary shaker set at 180 rpm. The 3-day-old cultures were subsequently used to obtain cell-free culture filtrate as previously described (Wonglom et al., 2019). Briefly, the PDB cultures were first filtered through Whatman filter paper Grade 44 using a vacuum filtration system and then filtered again through a 0.20-μm cellulose acetate syringe filter to obtain *T. hamatum* cell-free culture filtrate. Mycelial disks (7 mm in diameter) from 5-day-old PDA cultures of *L. theobromae* were placed in the center of Petri dishes (90 mm in diameter) containing 20 ml of PDA amended with different concentrations of the cell-free culture filtrate (0 [control], 0.5, 1, 5, and 10% v/v) and incubated at 25°C. The radial growth of *L. theobromae* was determined by measuring colony diameter after 48 h of incubation on the PDA plates.

A confrontation culture assay was conducted in a Petri dish with two-sections to determine the presence of antifungal activity of the volatiles produced by *T. hamatum*. Mycelial disks (7 mm in diameter) from a 5-day-old PDA culture of *T. hamatum* and *L. theobromae* were separately placed at the center of each section of the PDA petri plate and incubated at 25°C (Mao et al., 2019). Mycelial plugs from 5-day-old PDA plates without *T. hamatum* were used as a control. Antifungal activity of *T. hamatum* volatiles against *L. theobromae* was assessed after 48 h of coincubation using the following formula: Percent inhibition by *T. hamatum* volatiles = [(the largest diameter of *L. theobromae* colony in the control plates – the largest diameter of *L. theobromae* colony in the treatment plates)/the largest diameter of *L. theobromae* colony in control plates] × 100. Three biological replicates were utilized in each assay and the assay was repeated three times.

### Biocontrol efficacy of *Trichoderma hamatum* against *Lasiodiplodia theobromae* L1 on macadamia leaves *in vitro*

A conidial suspension of *T. hamatum* (1 × 10^6^ spores/ml) was sprayed evenly on the upper surface of fully expanded macadamia leaves that had been removed from macadamia plants. The leaves were air-dried and then two wounds were made on each leaf using sterilized needles. Each of the wounds on each wounded leaf was subsequently inoculated by placing a mycelial disk (5 mm in diameter) of *L. theobromae* over the wound site. Leaves sprayed with *T. hamatum* and inoculated with blank PDA disk without *L. theobromae* served as a positive (healthy) control, while leaves that were sprayed with sterilized water, wounded, and then inoculated with *L. theobromae* served as a negative (disease) control. All of the treated leaves were incubated on sterilized wet filter papers in Petri dishes for 4 days, after which average lesion area on each wound was determined. Three biological replicates (10 leaves for each replicate) were used in each assay and the assay was repeated three times.

### Biocontrol efficacy of *Trichoderma hamatum* against *Lasiodiplodia theobromae* on macadamia plants

Three-year-old potted macadamia plants ranging between 1 and 1.3 m in height were used in the biocontrol assay. The plant trunk (3 cm above the ground soil) of each plant was wounded with a 5-mm punch and injected with 5-ml of a conidial suspension (1 × 10^6^ spores/ml) of *T. hamatum*, and after air drying, subsequently inoculated with mycelial disks (5 mm in diameter) of *L. theobromae.* Each wound was covered with a wet, sterilized piece of cloth and sealed with plastic wrap. Wounded plants inoculated with 5-ml conidial suspension of *T. hamatum* (1 × 10^6^ spores/ml) and sterile PDA disks served as a positive (healthy) control, while wounded plants inoculated with 5-ml sterilized water and mycelial disks of *L. theobromae* served as a negative (disease) control. Three biological replicates (five plants for each replicate) were used in each assay and the assay was repeated three times. Disease severity was assessed at 20 days after inoculation using the following disease lesion scale: 0 = no spots, 1 = spot area 0–20%, 2 = 20–40%, 3 = 40–60%, 4 = 60–80% with 50% of the spots coalesced 50%, 5 = 80–100% with 75% of the spots coalesced. The disease lesion scale scores were converted to a disease severity index (DSI) using the following formula (Promwee et al., 2017):


DSI(%)=Σ(Scale×numberofleaves)/[(Maximumlevel)×(Totalnumberofleaves)]×100

### Statistical analysis

**FIGURE 2:**
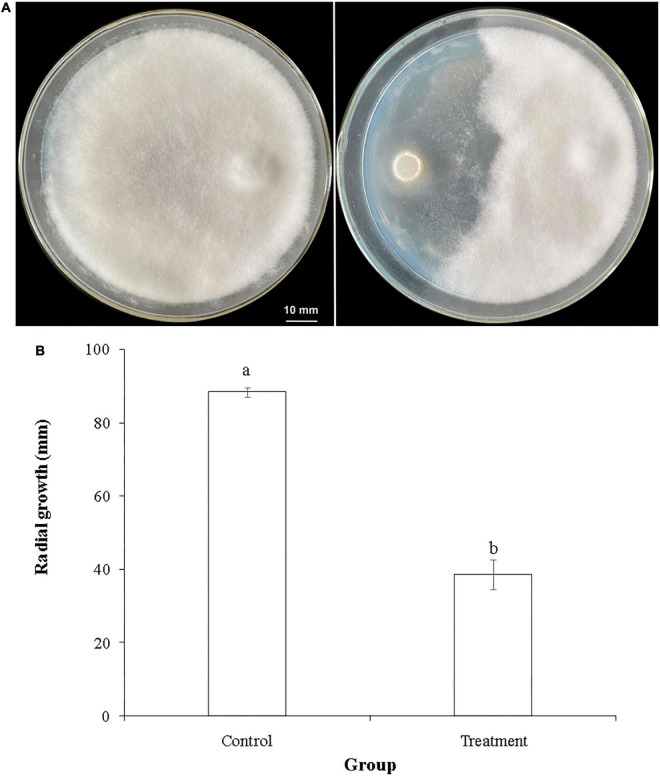
Inhibitory activity of *T. hamatum* against *L. theobromae* in a dual confrontation assay. **(A)** Representative photo of the radial growth of *L. theobromae* in the absence of (left panel) or presence of (right panel) *T. hamatum*. **(B)** Quantitative assessment of radial growth (PIRG) of *L. theobromae* in the absence (control) or presence (treatment) of *T. hamatum*. Different letters above each column indicate a significant difference (*P* < 0.05) between control and treatment groups according to Student’s *t*-test. Data represent the mean ± SD (*n* = 9).

**FIGURE 3:**
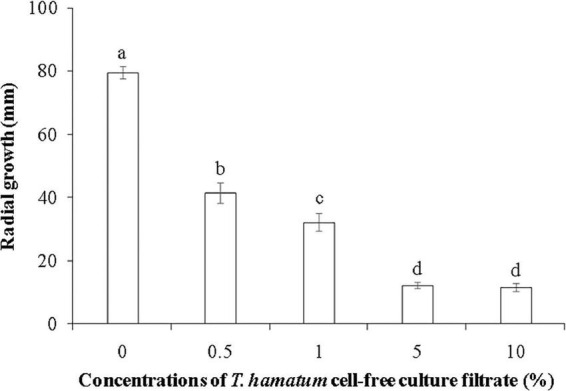
The effect of different concentrations of *T. hamatum* cell-free culture filtrate on the radial growth of *L. theobromae*. *L. theobromae* was grown on PDA medium amended with various concentrations of *T. hamatum* cell-free culture filtrate. Radial growth measurements (mm) were taken after 48 h of culture. Different letters above each column indicate a significant difference (*P* < 0.05) according to Duncan’s multiple range test. Data represent the mean ± SD (*n* = 9).

**FIGURE 4:**
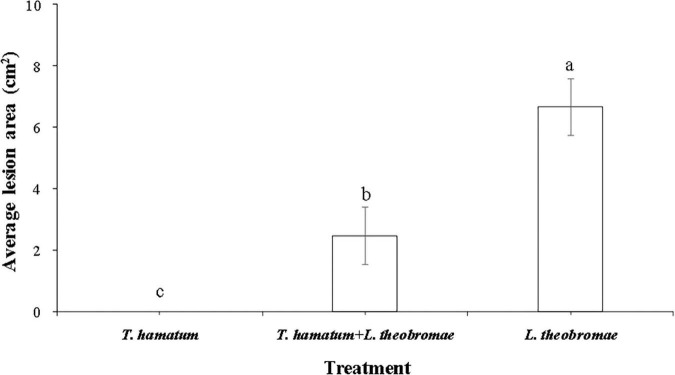
The quantitative data of lesion areas caused by *L. theobromae* on macadamia leaves 4 days after inoculation for the three treatment groups. (I) Leaves treated only with *T. hamatum* (healthy control); (II) Leaves treated with *T. hamatum* + *L. theobromae*; (III) Leaves treated only with *L. theobromae* (disease control). Different letters above each column indicate a significant difference (*P* < 0.05) according to Duncan’s multiple range test. Data represent the mean ± SD (*n* = 9).

**FIGURE 5:**
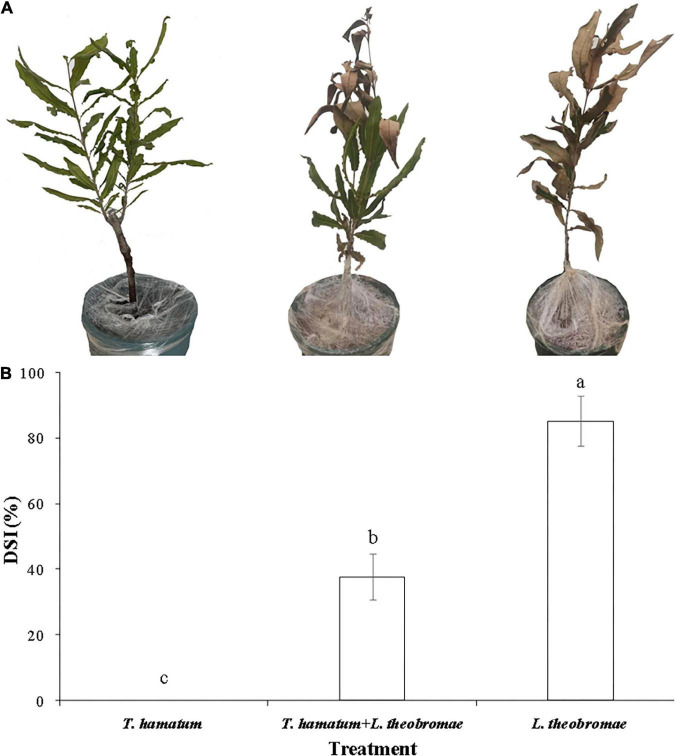
Disease symptoms and disease severity index (DSI) of potted macadamia plants infected with *L. theobromae* 20 days after inoculation. **(A)** Representative photos of potted, three-year-old macadamia trees untreated or treated with a conidial suspension of *T. hamatum* and then inoculated or non-inoculated with *L. theobromae*. Photos from left to right represent plants treated only with *T. hamatum* (healthy control), plants treated with *T. hamatum* + *L. theobromae*, and plants treated only with *L. theobromae* (disease control). **(B)** The corresponding disease severity index (DSI) of the three treatment groups. Different letters above each column indicate a significant difference (*P* < 0.05) according to a Duncan’s multiple range test. Data represent the mean ± SD (*n* = 9).

All statistical analyses were performed using SPSS version 20.0 (SPSS Inc., United States) software. Data with a single variable (treatment) were analyzed by a one-way ANOVA. Mean separations in Figure 2 were performed using a Student’s *t*-test, while mean separations in Figures 3–5 were performed using a Duncan’s multiple range test. Differences at *P* < 0.05 were considered significant. Data presented were pooled across three independent repeated experiments. As the experiment was not a significant variable, the statistical analyses were conducted on the pooled data (*n* = 9).

## Results and discussion

*Trichoderma hamatum* has been reported to have biocontrol activity against several fungal plant pathogens, including *Sclerotinia* spp. (Rabeendran et al., 2006), *Fusarium oxysporum* (Mao et al., 2020), *Rhizoctonia solani*, and *Pythium ultimum* (Lewis et al., 1996). The genome of *T. hamatum* GD12 has been sequenced and has provided fundamental information for studying its beneficial traits (Studholme et al., 2013). Results of the dual confrontation assay conducted in the present study indicated that *T. hamatum* had a significant inhibitory effect against *L. theobromae*, one of the major fungal pathogens of macadamia (Figure 2A). The radial growth of *L. theobromae* was significantly inhibited by *T. hamatum* (Figure 2B), exhibiting a percent inhibition of 56.3%. We hypothesized that non-volatile metabolites and/or volatiles produced by *T. hamatum* might contribute to its inhibitory activity against *L. theobromae*. Therefore, we assessed the inhibitory activity of cell-free culture filtrate and volatiles against *L. theobromae.*

Cell-free culture filtrates of *Trichoderma* spp., including *T. hamatum*, have been reported to have antifungal properties (Reino et al., 2008; Baiyee et al., 2019; Baazeem et al., 2021). Padder and Sharma (2011) reported that culture filtrate of *T. hamatum* had a significant inhibitory effect on spore germination of *Colletotrichum lindemuthianum*, and the inhibitory activity of culture filtrates of *Trichoderma* sp. have also been reported against other fungal pathogens, including *Fusarium solani* (Dugassa et al., 2021) and *F. oxysporum* (Shanmugam et al., 2008). In the current study, the cell-free culture filtrate of *T. hamatum* at a concentration ranging from 0.5 to 10% markedly inhibited the mycelial growth of *L. theobromae*, with inhibitory activity increasing with concentration. No difference in inhibitory activity was observed, however, between the 5 and 10% concentrations of cell-free culture filtrate (Figure 3).

Volatiles produced by *Trichoderma* spp. may also contribute to the inhibitory activity displayed against fungal pathogens (Li et al., 2018; Guo et al., 2019). Results of our culture assays support this premise. The culture assay utilizing Petri dishes with two separate sections clearly indicated that the volatiles produced by *T. hamatum* had a significant inhibitory effect on the growth of *L. theobromae*, with the percentage inhibition reaching 32.4%. The volatiles produced by *Trichoderma* species have been reported to include sesquiterpenes, diterpenes, and tetraterpenes (Lee et al., 2016), with *Trichoderma* species differing in the profile of the volatiles they produce based on specific fungal interactions (Guo et al., 2019). Therefore, the specific volatiles produced by *T. hamatum* in the presence of *L. theobromae* and the contribution of specific volatiles to the inhibitory activity need to be further investigated.

*Lasiodiplodia theobromae* can cause cankers on the trunks of macadamia tree and shoot necrosis (Fischer et al., 2017). In the present study, we found that *L. theobromae* can also cause lesions on macadamia leaves. When leaves were sprayed with a conidial suspension of *T. hamatum* (1 × 10^6^ spores/ml), however, the lesion area resulting from *L. theobromae* infection significantly decreased, relative to leaves treated only with *L. theobromae* (Figure 4). At present, there is limited information on the biocontrol efficacy of *Trichoderma* species against macadamia leaf diseases. We speculate that the non-volatile and/or volatile antifungal metabolites (Figures 2, 3) produced by *T. hamatum* may contribute to its biocontrol aptitude. The efficacy of biocontrol agents *in vitro*, however, does not guarantee their efficacy *in planta* (Collinge et al., 2022). Therefore, we also assessed the biocontrol efficacy of *T. hamatum* against *L. theobromae* on potted, three-year-old, macadamia plants. Macadamia trees are susceptible to a variety of fungal pathogens (Akinsanmi et al., 2016b; Wrona et al., 2020) and insects (Khun et al., 2021), which can result in significant economic losses. Studies on the biocontrol of insect have been significantly more numerous than studies on the biocontrol of plant diseases (Gutierrez-Coarite et al., 2018; Polaszek et al., 2020). In our present study, *T. hamatum* exhibited a high level of biocontrol efficacy against *L. theobromae* on three-year-old, potted macadamia trees (Figure 5A). The application of a conidial suspension (1 × 10^6^ spores/ml) of *T. hamatum* to wounds on the main stem of macadamia trees significantly decreased the DSI of *L. theobromae* from 85.1 to 37.7% (Figure 5B), indicating the good potential for use of *T. hamatum* as a biocontrol agent for the management of fungal diseases on macadamia.

## Conclusion

The present study demonstrated that *T. hamatum* can inhibit the growth of *L. theobromae in vitro*, and decrease lesion size on detached leaves, and disease severity on potted, three-year-old macadamia plants. Our study also indicates that non-volatile and volatile metabolites of *T. hamatum* may contribute to its inhibitory properties. Further, detailed studies on the mechanisms responsible for biocontrol activity, however, are needed. In particular, other potential modes of action of *T. hamatum* against *L. theobromae*, such as the induction of disease resistance and mycoparasitism, may also contribute and need to be investigated.

## Data availability statement

The original contributions presented in this study are included in the article/supplementary material, further inquiries can be directed to the corresponding authors.

## Author contributions

XiL, JLe, LY, JLi, and YS: conceptualization. XiL and YS: project administration. HB: resources. JLe and XuL: data curation. XiL, LY, JLi, and YS: writing – original draft. MW, LY, JLi, and YS: writing – review and editing. All authors contributed to the article and approved the submitted version.
